# Residential traffic exposure and pregnancy-related outcomes: a prospective birth cohort study

**DOI:** 10.1186/1476-069X-8-59

**Published:** 2009-12-22

**Authors:** Edith H van den Hooven, Vincent WV Jaddoe, Yvonne de Kluizenaar, Albert Hofman, Johan P Mackenbach, Eric AP Steegers, Henk ME Miedema, Frank H Pierik

**Affiliations:** 1 The Generation R Study Group, Erasmus Medical Center, Rotterdam, The Netherlands; 2 Department of Environment and Health, Netherlands Organisation for Applied Scientific Research (TNO), Delft, The Netherlands; 3 Department of Epidemiology, Erasmus Medical Center, Rotterdam, The Netherlands; 4 Department of Paediatrics, Erasmus Medical Center, Rotterdam, The Netherlands; 5 Department of Public Health, Erasmus Medical Center, Rotterdam, The Netherlands; 6 Department of Obstetrics and Gynaecology, Erasmus Medical Center, Rotterdam, The Netherlands

## Abstract

**Background:**

The effects of ambient air pollution on pregnancy outcomes are under debate. Previous studies have used different air pollution exposure assessment methods. The considerable traffic-related intra-urban spatial variation needs to be considered in exposure assessment. Residential proximity to traffic is a proxy for traffic-related exposures that takes into account within-city contrasts.

**Methods:**

We investigated the association between residential proximity to traffic and various birth and pregnancy outcomes in 7,339 pregnant women and their children participating in a population-based cohort study. Residential proximity to traffic was defined as 1) distance-weighted traffic density in a 150 meter radius, and 2) proximity to a major road. We estimated associations of these exposures with birth weight, and with the risks of preterm birth and small size for gestational age at birth. Additionally, we examined associations with pregnancy-induced hypertension, (pre)eclampsia, and gestational diabetes.

**Results:**

There was considerable variation in distance-weighted traffic density. Almost fifteen percent of the participants lived within 50 m of a major road. Residential proximity to traffic was not associated with birth and pregnancy outcomes in the main analysis and in various sensitivity analyses.

**Conclusions:**

Mothers exposed to residential traffic had no higher risk of adverse birth outcomes or pregnancy complications in this study. Future studies may be refined by taking both temporal and spatial variation in air pollution exposure into account.

## Background

Exposure to air pollution has been suggested to adversely affect various birth outcomes. As reported in a number of reviews, outcomes such as low birth weight, intrauterine growth restriction, and preterm birth have been associated with ambient air pollution levels, although effects were not always consistent between studies [1-4]. In large studies, assessing individual exposure to air pollution is often rather demanding for participants and requires extensive resources. Therefore, other approaches have been used to estimate exposure of individuals. Most studies have assessed exposure to air pollution using (an often limited number of) outdoor monitoring stations, either by using the station closest to the mother's home address at time of delivery [5,6], or by taking averaged concentrations measured at one or multiple monitor sites in a district [7,8]. Although concentrations of pollutants measured by ambient monitors may correspond to air pollution exposure at regional levels, this may not represent individual exposure [8], particularly for primary pollutants which display higher spatial heterogeneity. This spatial variation in air pollutant concentrations in urban areas, which can be largely attributed to traffic emissions, has been documented for several pollutants, such as nitrogen dioxide, black smoke, elemental carbon, ultrafine particles, and particulate matter (PM_2.5 _and PM_10_) [9,10]. Levels of these pollutants are elevated near roads [9,11,12], and are correlated with the traffic intensity on these roads [11,13]. Therefore, intra-urban gradients need to be taken into account.

Indicators of residential proximity to traffic, such as distance to a major road and traffic intensity around a location, are increasingly being used as proxies for long-term exposure to traffic pollutants. Epidemiological studies have linked these indicators to various health outcomes, such as respiratory symptoms [14,15], cardiovascular diseases [16], mortality rates [17] and childhood cancer [18]. In addition, few studies explored the effects of these indicators on birth and pregnancy outcomes. Associations of proximity to traffic with birth weight [19] and with the risks of preterm birth [20-23], small size for gestational age at birth [19,20,24], and low birth weight [20,21,24] have been suggested. Previous studies generally obtained information on birth outcomes from birth certificates. This may have reduced their ability to adjust for confounding, as birth records usually include limited information on potential confounding factors [8,25]. A prospective pregnancy cohort study with detailed exposure and covariate information can overcome this limitation [26,27].

Several potential biological mechanisms have been described through which air pollution could influence pregnancy outcomes, such as the induction of inflammation (placental, pulmonary, or systemic) and oxidative stress [28], eventually resulting in suboptimal placentation [7] and increased maternal susceptibility to infections [27]. These alterations could lead to adverse birth outcomes and maternal pregnancy complications such as pregnancy-induced hypertension and preeclampsia.

The aim of the present study was to investigate whether residential proximity to traffic is associated with various birth and pregnancy outcomes in a large population-based cohort study.

## Methods

### Design

The present study was embedded in the Generation R Study, a population-based prospective cohort study from pregnancy onwards. The Generation R study is designed to identify early environmental and genetic determinants of growth, development and health and has been described previously in detail [29,30]. In brief, the cohort includes mothers and children of different ethnicities living in Rotterdam, the Netherlands. Ideally, enrolment in the study took place in early pregnancy (gestational age <18 weeks), but was possible until the birth of the child. Out of the total number of eligible children in the study area, 61 percent participated in the study at birth. In total, 8,880 pregnant women with a delivery date between April 2002 and January 2006 entered the prenatal part of the study. The majority of these mothers (75%) was enrolled in early pregnancy (gestational age <18 weeks); 22 percent enrolled in mid-pregnancy (gestational age 18-24 weeks), and 3 percent enrolled in late pregnancy (gestational age >25 weeks) [30]. Data on pregnancy were collected on the basis of physical examinations, fetal ultrasounds, hospital registrations and questionnaires. Assessments were planned for early pregnancy, mid-pregnancy, and late pregnancy, but the individual time schemes depended on the specific gestational age at enrolment [29]. The study protocol was approved by the Medical Ethical Committee of Erasmus Medical Centre, Rotterdam. Written informed consent was obtained from all participants.

### Traffic exposure measures

Individual traffic exposure estimates at each participant's home address were assessed using Geographical Information Systems (GIS). The following traffic variables were used: 1) distance-weighted traffic density (DWTD) within a 150 meter radius around the home, and 2) proximity to a major road (with >10,000 vehicles/24 h). Input for the traffic exposure calculations was obtained from the local authorities of Rotterdam and included detailed digital maps with information on geographic locations and traffic characteristics for roads in the study area. The digital road maps include highways, arterial roads, main streets, and principal residential streets; the smallest local roads are not included. However, the traffic on these streets contributes only minorly to the total traffic flow in the area, and is therefore believed not to impact our traffic exposure measures. Annual average daily traffic intensities for the year 2004 were attached as attributes to the road segments for a dense network of roads. This data was used to estimate exposure for all pregnancies between 2002 and 2006. Based on index numbers for traffic intensity in the years 2002-2006 [31], it was concluded that the 2004 data could reasonably be applied to adjacent years. We geocoded the mothers' home addresses at time of delivery using ArcGIS (v9, ESRI). All matches were made at the address level. We constructed a 150 m radius buffer around the home. Distance-weighted traffic density was calculated using MapInfo Professional (v9.0, Pitney Bowes). To estimate the dispersion of motor vehicle exhaust, we employed a model that was based on a Gaussian distribution that assumes that 96% of the emitted pollutants disperse up to 150 m from the road:(1)

where D_*i *_is the distance from the road segment *i*. This curve was used to weigh the products of the length (in m) and the traffic intensities (in vehicles/24 h) of all road segments within the buffer. The weighted values were summed for each subject to obtain the distance-weighted traffic density. As vehicles may use multiple segments in the buffer, the DWTD values can be relatively high (up to millions of vehicles/24 h*m). Various definitions of DWTD are being used in the literature. We remark that our method to define DWTD is derived from the method used by Wilhelm & Ritz [21], with the difference that we take into account the length of the roads within the buffer. In addition to DWTD, we identified the nearest major road (with >10,000 vehicles/24 h) and calculated the distance to this road, up to a distance of 500 m.

### Birth and pregnancy outcomes

In mothers who were enrolled in early or mid-pregnancy, gestational age was established on the basis of fetal ultrasound examination during the first ultrasound visit, as the use of the last menstrual period (LMP) has several limitations [32]. In mothers who were enrolled in late pregnancy, the LMP was used for pregnancy dating. Medical records completed by midwives and obstetricians were used to obtain information about date of birth, birth weight, fetal sex, and occurrence of pregnancy complications. Main birth outcomes were birth weight (grams), small size for gestational age (SGA) at birth (<-2.0 SDS birth weight) and preterm birth (gestational age <37 weeks). Gestational age-adjusted standard deviation birth weight scores were based on published reference charts from a North European birth cohort [33], which are based on a large population and include the extremes of the birth weight distribution. Information about maternal pregnancy complications was available, including gestational diabetes mellitus, pregnancy-induced hypertension, and (pre)eclampsia. This last group consisted of women with eclampsia, preeclampsia or hemolysis elevated liver enzymes and low platelets (HELLP) syndrome. Gestational diabetes was diagnosed according to Dutch midwifery and obstetric guidelines using the following criteria: random glucose level >11.0 mmol/L, fasting glucose >7.0 mmol/l or a fasting glucose between 6.1 and 6.9 mmol/L with a subsequent abnormal glucose tolerance test, in women with no pre-existing diabetes. Pregnancy-induced hypertension was defined according to criteria described by the International Society for the Study of Hypertension in Pregnancy (ISSHP): development of systolic blood pressure ≥ 140 mmHg and/or diastolic blood pressure ≥ 90 mmHg without proteinuria after 20 weeks of gestation in previously normotensive women [34]. Preeclampsia was defined as development of systolic blood pressure ≥ 140 mmHg and/or diastolic blood pressure ≥ 90 mmHg after 20 weeks of gestation in a previously normotensive woman and proteinuria (defined as two or more dipstick readings of 2+ or greater, one catheter sample reading of 1+ or greater, or a 24 h urine collection containing at least 300 mg of protein) [34].

### Covariates

Based on previous studies, the following variables were considered as potential confounders: maternal age at intake, maternal educational level, maternal ethnicity, maternal body mass index (BMI), parity, maternal smoking, maternal alcohol consumption, and fetal sex. Information on maternal age, educational level, ethnicity, and parity was obtained in the first questionnaire at enrolment in the study. The highest educational level achieved by mother was used as an indicator of maternal socioeconomic status (SES) and was reclassified into three categories: (1) no education or primary school, (2) secondary school, and (3) higher education. Parity was classified into two categories: (1) nulliparous and (2) multiparous. Ethnic background of the woman was assessed on the basis of country of birth of her and her parents [30] and reclassified into five categories: (1) Dutch and Caucasian, (2) Turkish, (3) Moroccan, (4) Surinamese, and (5) other. Maternal anthropometrics were assessed at time of enrolment and at subsequent visits. Since the correlation of prepregnancy weight obtained by questionnaire and weight measured at enrolment was high (0.97, p < 0.001) [35], BMI was calculated on the basis of maternal weight and height at intake. Maternal smoking and alcohol consumption habits were assessed on the basis of three questionnaires (in early, mid-, and late pregnancy) by asking women whether they smoked/used alcohol before or during pregnancy (no/until pregnancy was known/yes). Mothers who reported in the first questionnaire that they did not smoke at all or had smoked until pregnancy was known, but reported smoking in the second or third questionnaire, were reclassified into the continued smoking category [36]. The same approach was followed for maternal alcohol consumption habits.

### Population for analysis

For the present analyses, data on all prenatally enrolled women were available (n = 8,880). We decided to restrict to participants living in the Northern part of Rotterdam at time of delivery, since participants living in the Southern part of Rotterdam did not participate in the postnatal follow-up study [30]. This yielded 7,506 women. We included all live singleton births (n = 7,431); women who gave birth to twins (n = 75) were excluded. We were able to calculate traffic exposure for 7,339 of these 7,431 women (99%) due to incomplete address data in 92 subjects. The associations between traffic indicators and pregnancy-related outcomes in mother and child were analyzed in the 7,339 remaining mothers.

### Statistical analysis

#### Main analyses

Based on a population for analysis of 7,000 subjects and a proportion exposed of 10%, we were able to detect a difference of 0.11 SD (type I error of 5%, type II error of 20% (power 80%)) for a continuous normally distributed outcome. Previous studies on air pollution and birth weight showed reductions in birth weight ranging to 140 grams, which is equal to 0.3 SD.

For the statistical analyses, distance-weighted traffic density was divided into quartiles. The distance to a major road was categorized as <50, 50-100, 100-150, 150-200, and >200 m. The associations of proximity to traffic with continuously measured birth weight were assessed using multivariate linear regression analyses. The associations between proximity to traffic and dichotomous birth and pregnancy outcomes were assessed using multivariate logistic regression analysis. Models were adjusted for known determinants of birth and pregnancy outcomes (maternal age, maternal ethnicity, maternal education, maternal BMI, parity, maternal smoking, and maternal alcohol consumption). Maternal age and BMI were included in the models as continuous variables. Models with birth weight as outcome were additionally adjusted for gestational age (with a linear term) and fetal sex. Models with SGA at birth and preterm birth were additionally adjusted for fetal sex. Additionally, we included indicator variables for month and year of birth in the models to control for season and long-term trends. Missing data on categorical factors were included in the analyses as a separate category.

#### Sensitivity analyses

We performed various sensitivity analyses to assess the robustness of our results.

First, to determine whether our findings were sensitive to the categorization of the traffic measures, we examined associations when using different cut-offs (e.g., the 80^th^, 90^th ^and 95^th ^percentiles of the distributions). Second, analyses were repeated when DWTD was calculated for different buffer radii. Next, to evaluate whether our results would change when we would introduce more contrast in our exposure levels, we calculated the distance to the nearest highway (with >25,000 vehicles/24 h) and examined associations with the main outcomes. Furthermore, to evaluate whether the results were sensitive to the method of determining gestational age (ultrasound versus LMP), we repeated the analyses after excluding women who were enrolled in late pregnancy, since only mothers who were enrolled in mid- and late pregnancy were dated on ultrasound. In addition, we repeated analyses in a subsample of women with data available on body mass index before pregnancy, and adjusted these analyses for BMI before pregnancy rather than BMI at intake. Furthermore, to evaluate whether our findings were sensitive to the definition of SGA at birth (which was based on reference charts for the North-European population), we repeated the analysis in a subcohort of Dutch participants only. Also, we investigated whether the associations between traffic exposure and pregnancy-related outcomes differed per educational level by performing stratified analyses. Finally, we conducted stratified analyses for residential mobility. We had information available on change of residence (yes/no/missing) in the period between seven months before conception and five months of pregnancy, and repeated the analyses for the different strata. All measures of association are presented with their 95% confidence intervals. Statistical analyses were performed using SPSS version 15.0 for Windows (SPSS Inc., Chicago, IL, USA).

## Results

### Subject characteristics

Table 1 shows baseline characteristics of the study population. The median age of the women was 30.5 years. The largest ethnic group was the Dutch and Caucasian (54.1%); other major ethnic groups were the Moroccan, Surinamese, and Turkish women. Of all women, 40.9% had completed high education. A total of 15.5% of the mothers smoked during pregnancy, and 36.5% continued using alcohol. Median gestational age at delivery was 40.1 weeks (90% range: 20.5-38.0); mean birth weight of the newborns was 3418 grams (SD 561). Of all children, 5.5% were born preterm and 3.5% were born small for gestational age. Among the pregnant women, 3.4% were diagnosed with pregnancy-induced hypertension, 2.0% developed (pre)eclampsia or HELLP, and 0.7% had gestational diabetes.

**Table 1:** Baseline characteristics (N = 7,339).

Maternal characteristics	
Age at intake (yr)	30.5 (20.5-38.0)
Weight at intake (kg)	67.0 (52.0-94.0)
Height (cm)	167.2 (7.4)
Body mass index at intake (kg/m^2^)	23.8 (19.3-33.5)
**Ethnicity**	
Dutch - Caucasian (%)	54.1
Turkish (%)	8.3
Moroccan (%)	6.4
Surinamese (%)	8.2
Other (%)	15.6
Missing (%)	7.4
**Educational level**	
No education/primary (%)	10.2
Secondary (%)	39.9
Higher (%)	40.9
Missing (%)	9.0
**Parity**	
Nulliparous (%)	55.0
Multiparous (%)	43.8
Missing (%)	1.2
**Smoking in pregnancy**	
No (%)	72.1
Yes (%)	15.5
Missing (%)	12.4
**Alcohol consumption in pregnancy**	
No (%)	52.7
Yes (%)	36.5
Missing (%)	10.8
**Birth and pregnancy outcomes**	
Gestational age at birth (wk)	40.1 (36.9-42.1)
Birth weight (g)	3417.6 (561.0)
SDS birth weight	-0.10 (1.03)
Male (%)	50.3
Small size for gestational age at birth (<-2.0 SDS) (%)	3.5
Preterm birth (<37 wk) (%)	5.5
Pregnancy-induced hypertension (%)	3.4
(Pre)eclampsia or HELLP (%)	2.0
Gestational diabetes (%)	0.7

Values are means (SDS) or medians (90% range) for variables with a skewed distribution, and percentages in case of categorical variables.

Of the total group, data were missing on maternal weight at intake (n = 31), maternal height at intake (n = 26), BMI at intake (n = 56), gestational age at birth (n = 2), fetal sex (n = 29), birth weight (n = 51), SDS birth weight (n = 61), SGA at birth (n = 61), preterm birth (n = 2), pregnancy-induced hypertension (n = 231), (pre)eclampsia/HELLP (n = 231), and gestational diabetes (n = 271).

### Traffic variables

**Figure 1:**
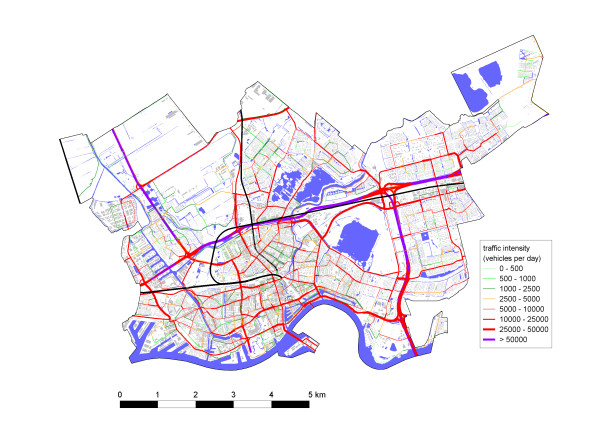
Map of the study area (Rotterdam North) showing the road network and traffic intensities (see legend), rail network (black lines), residences (in grey), and surface water (in blue).

A map of the study area showing the road network, traffic intensities, and residences is shown in figure 1. Characteristics of the distributions of distance-weighted traffic density and distance to a major road are shown in Additional file 1. The distribution of DWTD was highly skewed, with a maximum 18,500,000 vehicles/24 h*m. In total, 14.5% of the participants lived within 50 m of a major road and 36.0% lived more than 200 m from a major road. The correlation between DWTD and distance to a major road was moderate (Spearman rho = 0.70). Distance to a highway was only weakly correlated to the other traffic variables (rho = -0.28 and 0.32). In analyses with maternal sociodemographic variables and proximity to traffic, we observed that low body mass index, high educational level, and nulliparity were positively associated with residential traffic exposure, whereas Moroccan women had lower exposure to residential traffic (results not shown).

### Proximity to traffic and birth and pregnancy outcomes

There were no substantial differences in traffic exposure between cases and non-cases of adverse birth outcomes or maternal pregnancy complications (results not shown). Crude associations between proximity to traffic and birth outcomes and maternal pregnancy complications are presented in Additional files 2 and 3. We observed a few significant associations between distance to a major road and birth weight, and between DWTD and preterm birth. No associations with pregnancy complications were detected. Tables 2 and 3 show the results of the linear and logistic regression analyses for associations between proximity to traffic and pregnancy-related outcomes in mother and child, adjusted for covariates. We observed significant associations between the second DWTD quartile and the risk for preterm birth, and between living 100 to 150 m from a major road and birth weight. Although not significant, there was some evidence of an exposure-response pattern for SGA at birth across the quartiles of DWTD and categories of distance to a major road. This pattern was less clear for birth weight and preterm birth. No consistent associations were observed between traffic exposure and pregnancy complications, although we did see a tendency towards higher odds ratios in the highest exposure categories.

**Table 2:** Covariate-adjusted associations between residential traffic exposure and birth outcomes.

	Birth weight (g) ^b^	**Small for gestational age **^c^ * (n of cases)*	**Preterm birth **^c ^ *(n of cases)*
**Distance-weighted traffic density **(veh/24 h*m) ^a^			
< 158,503	*Reference*	*Reference (n = 61)*	*Reference (n = 84)*
158,503 - 546,770	-20 (-47, 8)	0.94 (0.65, 1.36) *(n = 60)*	1.37 (1.02, 1.84) * *(n = 112)*
546,770 - 1,235,384	-9 (-37, 18)	0.99 (0.69, 1.43) *(n = 62)*	1.33 (0.98, 1.79) † *(n = 110)*
> 1,235,384	6 (-21, 34)	1.12 (0.78, 1.59) *(n = 74)*	1.18 (0.87, 1.59) *(n = 100)*
**Distance to major road **(m)			
> 200 *(n = 2646)*	*Reference*	*Reference (n = 82)*	*Reference (n = 134)*
150-200 *(n = 1066)*	-21 (-52, 9)	1.00 (0.67, 1.49) *(n = 38)*	1.09 (0.79, 1.50) *(n = 59)*
100-150 *(n = 1258)*	-41 (-69, -12) *	1.01 (0.69, 1.48) *(n = 44)*	1.13 (0.84, 1.52) *(n = 75)*
50-100 *(n = 1302)*	8 (-20, 37)	1.12 (0.78, 1.62) *(n = 51)*	1.08 (0.80, 1.45) *(n = 74)*
0-50 *(n = 1067)*	-6 (-36, 24)	1.14 (0.77, 1.68) *(n = 42)*	1.15 (0.84, 1.58) *(n = 64)*

* p < 0.05

† p < 0.10

^a ^Values listed are the <25^th^, 25-50^th^, 50-75^th ^and >75^th ^percentiles of the DWTD values.

^b ^Values are regression coefficients (95% confidence interval) and reflect the difference in birth weight for change in traffic parameters. Analyses are based on 7,288 subjects. The model is adjusted for gestational age, fetal sex, maternal age, maternal education, maternal ethnicity, maternal body mass index, parity, maternal smoking, maternal alcohol consumption, month of birth, and year of birth.

^c ^Values are odds ratios (95% confidence interval) and reflect the risk for adverse birth outcomes for change in traffic parameters. Analyses are based on 7,278 subjects for small for gestational age at birth and 7,337 subjects for preterm birth. Models are adjusted for fetal sex, maternal age, maternal education, maternal ethnicity, maternal body mass index, parity, maternal smoking, maternal alcohol consumption, month of birth, and year of birth.

**Table 3:** Covariate-adjusted associations between residential traffic exposure and pregnancy complications.

	**Pregnancy-induced hypertension **^b^ *(n of cases)*	**(Pre)eclampsia or HELLP **^b^ *(n of cases)*	**Gestational diabetes **^b^ *(n of cases)*
**Distance-weighted traffic density **(veh/24 h*m) ^a^			
< 158,503	*Reference (n = 64)*	*Reference (n = 34)*	*Reference (n = 15)*
158,503 - 546,770	1.00 (0.69, 1.45) *(n = 59)*	0.94 (0.57, 1.55) *(n = 31)*	0.69 (0.30, 1.57) *(n = 10)*
546,770 - 1,235,384	0.90 (0.62, 1.30) *(n = 59)*	1.12 (0.70, 1.79) *(n = 39)*	1.07 (0.51, 2.23) *(n = 15)*
> 1,235,384	1.07 (0.75, 1.53) *(n = 68)*	1.14 (0.71, 1.82) *(n = 40)*	0.79 (0.35, 1.81) *(n = 10)*
**Distance to major road **(m)			
> 200 *(n = 2646)*	*Reference (n = 93)*	*Reference (n = 54)*	*Reference (n = 19)*
150-200 *(n = 1066)*	0.88 (0.57, 1.36) *(n = 29)*	0.74 (0.42, 1.29) *(n = 17)*	1.07 (0.47, 2.44) *(n = 9)*
100-150 *(n = 1258)*	0.94 (0.64, 1.39) *(n = 39)*	0.96 (0.59, 1.56) *(n = 25)*	0.77 (0.32, 1.88) *(n = 7)*
50-100 *(n = 1302)*	1.07 (0.75, 1.54) *(n = 49)*	0.85 (0.52, 1.38) *(n = 24)*	1.13 (0.51, 2.50) *(n = 10)*
0-50 *(n = 1067)*	1.08 (0.74, 1.60) *(n = 40)*	1.03 (0.63, 1.69) *(n = 24)*	0.68 (0.25, 1.86) *(n = 5)*

^a ^Values listed are the <25^th^, 25-50^th^, 50-75^th ^and >75^th ^percentiles of the DWTD values.

^b ^Values are odds ratios (95% confidence interval) and reflect the risk for pregnancy complications for change in traffic parameters. Analyses are based on 7,108 subjects for pregnancy-induced hypertension and for (pre)eclampsia or HELLP, and on 7,068 subjects for gestational diabetes. Models are adjusted for maternal age, maternal education, maternal ethnicity, maternal body mass index, parity, maternal smoking, maternal alcohol consumption, month of birth, and year of birth.

### Sensitivity analyses

Although we observed a few significant associations between proximity to traffic and birth outcomes (Table 2), these could not be reproduced in sensitivity analyses when different categorizations and buffer radii for the traffic measures were chosen. In line with the results from the main analyses, no associations with pregnancy outcomes were observed in the sensitivity analyses. Similarly, when distance to a highway was used as an exposure metric, no significant associations with birth and pregnancy outcomes were observed. Furthermore, results of the analyses did not change after excluding women who were enrolled in late pregnancy and for whom gestational age was determined based on LMP. Results were also comparable when analyses were adjusted for maternal BMI before pregnancy rather than maternal BMI at intake. In addition, logistic regression analysis with SGA at birth in the subgroup of Dutch participants yielded similar results (see Additional file 4). Stratified analyses by educational level did not show different results (see Additional files 5, 6 and 7 for associations between proximity to traffic and selected outcomes). However, it must be noted that the statistical power for some of these analyses was limited due to small numbers of participants in the subgroups, especially in the lowest educational group. Finally, stratified analyses by residential mobility showed that results were not different across strata (see Additional files 8 and 9).

## Discussion

To our knowledge, this is the first report on residential proximity to traffic and pregnancy-related outcomes that was based on a prospective cohort study. A significant number of examinations were performed in mothers and children, providing information on the relevant potentially confounding variables. We observed no associations between residential proximity to traffic and birth and pregnancy outcomes, also after controlling for potential confounders. Proximity to traffic was defined by means of two variables: distance-weighted traffic density (DWTD) and distance to a major road. These traffic measures are used to capture the spatial variation within a city, and have been applied previously in a small number of register-based studies on birth outcomes [19-24]. These studies did not show conclusive evidence. Two studies, both conducted in California, used distance-weighted traffic density as an exposure metric. The first study observed an association between increased DWTD and the risk of preterm birth, with stronger effects in women living in lower SES areas [21]. The second study reported that DWTD was positively associated with the risk of preterm birth in mothers living in low SES neighbourhoods whose third trimester fell during winter, and in mothers living in moderate SES neighbourhoods [23]. Another study, conducted in Massachusetts, used cumulative traffic density as an exposure metric, which is a more rough exposure metric than DWTD as the products of the traffic intensities and the lengths of the road segments are not weighted. That study observed an association for cumulative traffic density with the risk for SGA at birth, but not with birth weight and preterm birth. Moreover, the researchers also reported evidence for effect modification by socioeconomic status, with stronger effects of proximity to traffic in low educated women and in women living in lower SES areas [19]. Distance to a major road or highway was examined previously in relation to birth outcomes as well. The Massachusetts study observed associations between distance to a primary highway and birth weight, but not with the risks of preterm birth and SGA at birth [19]. In Taiwan, an increased risk of preterm delivery was reported in mothers living within 500 m of one particular freeway compared to mothers living between 500-1500 m from this freeway [22]. Two recent studies in British Columbia produced different findings. Brauer et al. (2008) observed an increased risk of SGA at birth in mothers living within 50 m from an expressway or highway (with a mean of >21,000 vehicles/day), but no association was found with the risk of preterm birth. Also, no significant associations were detected for those living within 50 or 150 m from a road with a mean of 15,000-18,000 vehicles/day [24]. The second study reported that proximity to a highway (with a minimum speed of 70 km/hr) was associated with preterm birth, but not with SGA at birth [20]. Moreover, a higher susceptibility among advantaged mothers was described, in contrast to the American studies. In our study, stratified analyses on SES showed no differences in susceptibility for traffic exposure between the SES groups.

To our knowledge, no previous studies have been conducted on residential proximity to traffic, or on air pollution exposure in the broader sense, and pregnancy complications. Recently, it has been suggested that studying these outcomes may provide insights into the underlying mechanisms [37]. In the present study, no crude or adjusted associations between residential proximity to traffic and pregnancy complications were observed, although we did observe tendencies towards elevated odds ratios in the highest exposure groups.

There are several differences between earlier studies and the present study that may explain the dissimilar findings. First of all, previous studies primarily relied upon birth records, which may have resulted in less complete information on important confounders. In our study, maternal education, ethnicity, body mass index, parity, and smoking were the main predictors in most of the models with birth outcomes, and in some of the models with pregnancy complications. Earlier studies did not have information on all of these covariates [20-24]. As a result, they may have been more susceptible to residual confounding, which could have affected some of the observed associations.

Secondly, the exposure metrics used in the different studies are based on different input data. Also, the classification of roads, calculation methods (e.g. buffer size), and the accuracy and completeness of traffic and road data may vary between studies.

Third, the observed differences between previous studies and the present study may be related to the geographic location of the studies. Rotterdam is the second largest city in the Netherlands and has a high population density. It is characterized by high emissions from road traffic, shipping, households, and industry. In the year 2004, average air pollutant levels derived from ambient monitoring stations in the Rijnmond region (the larger Rotterdam-area) were 30.7 μg/m^3 ^for PM_10_, 43.8 μg/m^3 ^for nitrogen dioxide (NO_2_), and 13.8 μg/m^3 ^for sulfur dioxide (SO_2_) [38]. These average concentrations are based on both regional background stations and traffic stations, consequently, pollutant levels in the specific (urban) area under study may be even higher. Previous studies on proximity to traffic and pregnancy outcomes have mainly been performed in the United States, Canada and Taiwan. No previous studies on these specific exposure measures and outcomes have been conducted in a European area, where air pollution may differ in terms of composition and concentrations.

This study has some potential limitations. First, there is the potential for misclassification of exposure. Exposure levels were estimated at the home address, whereas pregnant women do not spend all of their time at home. No detailed information was available about time-activity patterns of the women. However, it has been suggested that outdoor levels of traffic components are well correlated with indoor levels [39,40] and are good predictors of personal exposure [39,41]. Furthermore, exposure misclassification may arise from a change in address during pregnancy. As residential mobility during pregnancy has previously been shown to be differential by sociodemographic factors (e.g., maternal age, household income, parity, and ethnicity) [42], it could influence the results of our study. In stratified analyses, we observed that results were not different across those who did/did not change residence in the period between seven months before conception and five months of pregnancy. This indicates that residential mobility did not have a large effect on our effect estimates.

Second, the sample size of our study was smaller than that of previous studies, which were based on birth certificate data and had sample sizes of 37,000-99,000 subjects. Our study had 7,339 participants. We were able to detect a difference of 0.11 SD in birth weight, which is smaller than effect sizes observed in previous studies. However, the power to detect a relationship between air pollution and some of the dichotomous outcome measures was lower compared to previous studies, especially for the analyses with pregnancy complications.

Furthermore, gestational age could not be determined based on ultrasound examinations in 3% of the participants, because they were enrolled in late pregnancy. Nevertheless, results were comparable when these women were included or excluded.

Another limitation is related to the traffic density measures. These are derived from digital maps that cover the most important residential roads, but do not include the smallest local roads. As a result, traffic on these streets is not counted in the distance-weighted traffic density.

Finally, traffic measures may be viewed as crude estimates of air pollution. They do not take into account influencing factors such as type of traffic, emission factors, meteorology, and land cover data. Furthermore, they are based on annual averages and do not reflect seasonal, monthly or daily differences in air pollution levels. Ideally, these temporal variations would be taken into account in the exposure assessment, next to the spatial variability. This has been done by a few earlier pregnancy studies, some of them conducted in Europe, in which air pollution concentrations were modeled and subsequently adjusted for temporal variation [24,25,43,44]. Unfortunately, we were not able to take into account temporal variations in our air pollution exposure assessment, but we are planning to do this for future analyses. Despite these limitations, a recent study that assessed the validity of traffic variables showed that measures of (weighted) traffic density can well be used as predictors of measured NO_2_, and are therefore good proxies for exposure to road traffic [45]. So, when direct measurements or modeled levels of traffic-related air pollutants are not available, traffic measures are a good alternative, as they are relatively simple measures that are easy to apply and to interpret [24].

## Conclusions

The present study is based on a prospective population-based cohort with a large number of subjects studied from early pregnancy onwards. Exposures were estimated at the individual level, and detailed individual information on relevant confounders was available. In the city of Rotterdam, residential proximity to traffic was not associated with birth and pregnancy outcomes, contrary to previous studies. Future studies are needed to further investigate this relationship, preferably with more detailed data on temporal and spatial variation in exposure.

## List of abbreviations

PM_10_: particulate matter with an aerodynamic diameter <10 μm; PM_2.5_: particulate matter with an aerodynamic diameter <2.5 μm; NO_2_: nitrogen dioxide; SO_2_: sulfur dioxide; DWTD: distance-weighted traffic density; LMP: last menstrual period; SGA: small size for gestational age; HELLP: hemolysis elevated liver enzymes and low platelets syndrome; BMI: body mass index; SES: socioeconomic status; SD: standard deviation.

## Competing interests

The authors declare that they have no competing interests.

## Authors' contributions

All authors have made substantial contribution to this study and to the writing and editing of the manuscript. Additional contributions are as follows: EHH was involved in the planning of the study, data collection, statistical analyses, and interpretation of data, and drafted the manuscript; FHP, VWVJ and YK contributed to the design of the study, supervision, interpretation of data and critical review of the manuscript; EAPS, HMEM and JPM had critical input into the manuscript; AH conceptionalised the Generation R study and participated in its design and conduction. All authors read and approved the final manuscript.

## Supplementary Material

Additional file 1 **Table S1. Distribution of traffic indicators**. The table presents the characteristics of the distributions (minimum, 25^th ^percentile, median, 75^th ^percentile, and maximum) of distance-weighted traffic density and distance to a major road in the population. Click here for file

Additional file 2 **Table S2. Crude associations between residential traffic exposure and birth outcomes**. The table shows the crude associations from linear and logistic regression analyses between proximity to traffic and birth outcomes. Click here for file

Additional file 3 **Table S3. Crude associations between residential traffic exposure and pregnancy complications**. The table shows the crude associations from logistic regression analyses between proximity to traffic and pregnancy complications. Click here for file

Additional file 4 **Table S4. Covariate-adjusted associations between residential traffic exposure and SGA at birth in Dutch children (n = 3,414)**. The table shows the results from the sensitivity analysis on SGA at birth in a subgroup of Dutch participants. Click here for file

Additional file 5 **Table S5. Covariate-adjusted associations between residential traffic exposure and birth weight, stratified for maternal education**. The table contains the results from the stratified analyses by educational level on the association between proximity to traffic and birth weight. Click here for file

Additional file 6 **Table S6. Covariate-adjusted associations between residential traffic exposure and SGA at birth, stratified for maternal education**. The table contains the results from the stratified analyses by educational level on the association between proximity to traffic and SGA at birth. Click here for file

Additional file 7 **Table S7. Covariate-adjusted associations between residential traffic exposure and pregnancy-induced hypertension, stratified for maternal education**. The table contains the results from the stratified analyses by educational level on the association between proximity to traffic and pregnancy-induced hypertension. Click here for file

Additional file 8 **Table S8. Covariate-adjusted associations between residential traffic exposure and birth outcomes in non-movers (n = 1,118)**. The table presents the results from the sensitivity analyses on birth outcomes in the subgroup of non-movers. Click here for file

Additional file 9 **Table S9. Covariate-adjusted associations between residential traffic exposure and pregnancy-complications in non-movers (n = 1,118)**. The table presents the results from the sensitivity analyses on pregnancy complications in the subgroup of non-movers. Click here for file
