# Supplementary material for: Residential traffic exposure and pregnancy-related outcomes: a prospective birth cohort study
Source: Environ Health. 2009 Dec 22;8:59. doi: 10.1186/1476-069X-8-59 (PMC2811104; doi:10.1186/1476-069X-8-59)
Supplement: Additional file 1 — Table S1. Distribution of traffic indicators. The table presents the characteristics of the distributions (minimum, 25th percentile, median, 75th percentile, and maximum) of distance-weighted traffic density and distance to a major road in the population. [file 1476-069X-8-59-S1.PDF]

Additional file 1. Table S1. Distribution of traffic indicators.

	**Minimum**	**25th percentile**	**Median**	**75th percentile**	**Maximum**
**Distance-weighted traffic density** (vehicles/24h*m)	0	$1.6 * 10^5$	$5.5 * 10^5$	$1.2 * 10^6$	$1.9 * 10^7$
**Distance to a major road** (m) [a]	7	74	143	225	498
