# Supplementary material for: Residential traffic exposure and pregnancy-related outcomes: a prospective birth cohort study
Source: Environ Health. 2009 Dec 22;8:59. doi: 10.1186/1476-069X-8-59 (PMC2811104; doi:10.1186/1476-069X-8-59)
Supplement: Additional file 2 — Table S2. Crude associations between residential traffic exposure and birth outcomes. The table shows the crude associations from linear and logistic regression analyses between proximity to traffic and birth outcomes. [file 1476-069X-8-59-S2.PDF]

Additional file 2. Table S2. Crude associations between residential traffic exposure and birth outcomes.

	**Birth weight** (g) [b]	**Small for gestational age** [c] *(n of cases)*	**Preterm birth** [c] *(n of cases)*
**Distance-weighted traffic density** (veh/24h*m) [a]			
< 158,503	*Reference*	*Reference (n=61)*	*Reference (n=84)*
158,503 – 546,770	-29 (-58, 0) †	0.99 (0.69, 1.42) *(n=60)*	1.37 (1.02, 1.84) * *(n=112)*
546,770 – 1,235,384	-19 (-48, 10)	1.02 (0.71, 1.47) *(n=62)*	1.36 (1.01, 1.83) * *(n=110)*
> 1,235,384	-14 (-43, 15)	1.22 (0.87, 1.73) *(n=74)*	1.25 (0.92, 1.69) *(n=100)*
**Distance to major road** (m)			
> 200 *(n=2646)*	*Reference*	*Reference (n=82)*	*Reference (n=134)*
150-200 *(n=1066)*	-48 (-79, -16) *	1.15 (0.78, 1.70) *(n=38)*	1.13 (0.82, 1.55) *(n=59)*
100-150 *(n=1258)*	-65 (-95, -35) **	1.13 (0.78, 1.65) *(n=44)*	1.20 (0.89, 1.61) *(n=75)*
50-100 *(n=1302)*	-18 (-48, 12)	1.27 (0.89, 1.81) *(n=51)*	1.16 (0.86, 1.56) *(n=74)*
0-50 *(n=1067)*	-28 (-59, 4) †	1.28 (0.88, 1.87) *(n=42)*	1.23 (0.90, 1.68) *(n=64)*

** p < 0.01

* p < 0.05

† p < 0.10

[a] *Values listed are the <25th, 25-50th, 50-75th and >75th percentiles of the DWTD values.*

[b] *Values are regression coefficients (95% confidence interval) and reflect the difference in birth weight for change in traffic parameters. Analyses are based on 7,288 subjects. Models are adjusted for gestational age and fetal sex.*

[c] *Values are odds ratios (95% confidence interval) and reflect the risk for adverse birth outcomes for change in traffic parameters. Analyses are based on 7,278 subjects for small for gestational age at birth and 7,337 subjects for preterm birth. Models are adjusted for fetal sex.*
