# Supplementary material for: Residential traffic exposure and pregnancy-related outcomes: a prospective birth cohort study
Source: Environ Health. 2009 Dec 22;8:59. doi: 10.1186/1476-069X-8-59 (PMC2811104; doi:10.1186/1476-069X-8-59)
Supplement: Additional file 3 — Table S3. Crude associations between residential traffic exposure and pregnancy complications. The table shows the crude associations from logistic regression analyses between proximity to traffic and pregnancy complications. [file 1476-069X-8-59-S3.PDF]

Additional file 3. Table S3. Crude associations between residential traffic exposure and pregnancy complications.

	**Pregnancy-induced hypertension** [b] *(n of cases)*	**(Pre)eclampsia or HELLP** [b] *(n of cases)*	**Gestational diabetes** [b] *(n of cases)*
**Distance-weighted traffic density** (veh/24h*m) [a]			
< 158,503	*Reference (n=64)*	*Reference (n=34)*	*Reference (n=15)*
158,503 – 546,770	0.92 (0.64, 1.32) *(n=59)*	0.91 (0.56, 1.50) *(n=31)*	0.66 (0.30, 1.48) *(n=10)*
546,770 – 1,235,384	0.92 (0.64, 1.32) *(n=59)*	1.15 (0.73, 1.84) *(n=39)*	1.00 (0.49, 2.05) *(n=15)*
> 1,235,384	1.06 (0.75, 1.50) *(n=68)*	1.18 (0.74, 1.87) *(n=40)*	0.67 (0.30, 1.49) *(n=10)*
**Distance to major road** (m)			
> 200 *(n=2646)*	*Reference (n=93)*	*Reference (n=54)*	*Reference (n=19)*
150-200 *(n=1066)*	0.77 (0.50, 1.17) *(n=29)*	0.78 (0.45, 1.34) *(n=17)*	1.17 (0.53, 2.60) *(n=9)*
100-150 *(n=1258)*	0.87 (0.60, 1.28) *(n=39)*	0.97 (0.60, 1.56) *(n=25)*	0.76 (0.32, 1.82) *(n=7)*
50-100 *(n=1302)*	1.07 (0.75, 1.52) *(n=49)*	0.89 (0.55, 1.45) *(n=24)*	1.07 (0.50, 2.31) *(n=10)*
0-50 *(n=1067)*	1.07 (0.73, 1.55) *(n=40)*	1.10 (0.68, 1.79) *(n=24)*	0.65 (0.24, 1.76) *(n=5)*

[a] *Values listed are the <25th, 25-50th, 50-75th and >75th percentiles of the DWTD values.*

[b] *Values are odds ratios (95% confidence interval) and reflect the risk for pregnancy complications for change in traffic parameters. Analyses are based on 7,108 subjects for pregnancy-induced hypertension and for (pre)eclampsia or HELLP, and on 7,068 subjects for gestational diabetes.*
