# Supplementary material for: Residential traffic exposure and pregnancy-related outcomes: a prospective birth cohort study
Source: Environ Health. 2009 Dec 22;8:59. doi: 10.1186/1476-069X-8-59 (PMC2811104; doi:10.1186/1476-069X-8-59)
Supplement: Additional file 4 — Table S4. Covariate-adjusted associations between residential traffic exposure and SGA at birth in Dutch children (n = 3,414). The table shows the results from the sensitivity analysis on SGA at birth in a subgroup of Dutch participants. [file 1476-069X-8-59-S4.PDF]

Additional file 4. Table S4. Covariate-adjusted associations between residential traffic exposure and SGA at birth in Dutch children (N=3,414).

	**Small for gestational age** [b] *(n of cases)*
**Distance-weighted traffic density** (veh/24h*m) [a]	
< 158,503	*Reference (n=27)*
158,503 – 546,770	0.82 (0.46, 1.46) *(n=22)*
546,770 – 1,235,384	1.08 (0.62, 1.87) *(n=28)*
> 1,235,384	0.74 (0.41, 1.34) *(n=21)*
**Distance to major road** (m)	
> 200	*Reference (n=42)*
150-200	0.52 (0.25, 1.08) † *(n=14)*
100-150	1.11 (0.64, 1.93) (*n=13)*
50-100	0.74 (0.39, 1.41) *(n=20)*
0-50	1.09 (0.58, 2.05) *(n=9)*

† p < 0.10

[a] *Values listed are the <25th, 25-50th, 50-75th and >75th percentiles of the DWTD values.*

[b] *Values are odds ratios (95% confidence interval) and reflect the risk for small for gestational age at birth for change in traffic parameters. Models are adjusted for fetal sex, maternal age, maternal education, maternal body mass index, parity, maternal smoking, maternal alcohol consumption, month of birth, and year of birth.*
