# Supplementary material for: Residential traffic exposure and pregnancy-related outcomes: a prospective birth cohort study
Source: Environ Health. 2009 Dec 22;8:59. doi: 10.1186/1476-069X-8-59 (PMC2811104; doi:10.1186/1476-069X-8-59)
Supplement: Additional file 5 — Table S5. Covariate-adjusted associations between residential traffic exposure and birth weight, stratified for maternal education. The table contains the results from the stratified analyses by educational level on the association between proximity to traffic and birth weight. [file 1476-069X-8-59-S5.PDF]

**Additional file 5. Table S5.** Covariate-adjusted associations between residential traffic exposure and birth weight, stratified for maternal education.

| | **Birth weight** (g) [b] | | |
|---|---|---|---|
| | **None/primary education** | **Secondary education** | **Higher education** |
| **Distance-weighted traffic density** (veh/24h*m) [a] | | | |
| < 158,503 | *Reference* | *Reference* | *Reference* |
| 158,503 – 546,770 | -26 (-115, 64) | -17 (-60, 26) | -20 (-63, 23) |
| 546,770 – 1,235,384 | -56 (-145, 32) | -20 (-63, 22) | 10 (-34, 55) |
| > 1,235,384 | 43 (-49, 134) | 0 (-44, 43) | 5 (-38, 47) |
| **Distance to major road** (m) | | | |
| > 200 | *Reference* | *Reference* | *Reference* |
| 150-200 | 7 (-85, 100) | -32 (-80, 17) | -20 (-68, 28) |
| 100-150 | -2 (-94, 90) | -50 (-94, -6) * | -45 (-91, 2) † |
| 50-100 | 32 (-57, 121) | -7 (-52, 37) | 16 (-29, 61) |
| 0-50 | 98 (-5, 201) † | -33 (-83, 16) | 5 (-41, 50) |

* p < 0.05

† p < 0.10

[a] *Values listed are the <25$^{th}$, 25-50$^{th}$, 50-75$^{th}$ and >75$^{th}$ percentiles of the DWTD values.*

[b] *Values are regression coefficients (95% confidence interval) and reflect the difference in birth weight for change in traffic parameters. Models are adjusted for gestational age, fetal sex, maternal age, maternal ethnicity, maternal body mass index, parity, maternal smoking, maternal alcohol consumption, month of birth, and year of birth.*
