# Supplementary material for: Residential traffic exposure and pregnancy-related outcomes: a prospective birth cohort study
Source: Environ Health. 2009 Dec 22;8:59. doi: 10.1186/1476-069X-8-59 (PMC2811104; doi:10.1186/1476-069X-8-59)
Supplement: Additional file 6 — Table S6. Covariate-adjusted associations between residential traffic exposure and SGA at birth, stratified for maternal education. The table contains the results from the stratified analyses by educational level on the association between proximity to traffic and SGA at birth. [file 1476-069X-8-59-S6.PDF]

Additional file 6. Table S6. Covariate-adjusted associations between residential traffic exposure and SGA at birth, stratified for maternal education.

	Small for gestational age [b]		
	**None/primary education**	**Secondary education**	**Higher education**
**Distance-weighted traffic density** (veh/24h*m) [a]			
< 158,503	*Reference*	*Reference*	*Reference*
158,503 – 546,770	1.11 (0.38, 3.27)	0.64 (0.36, 1.16)	1.20 (0.64, 2.26)
546,770 – 1,235,384	1.03 (0.35, 3.03)	0.95 (0.56, 1.62)	1.05 (0.53, 2.07)
> 1,235,384	0.54 (0.17, 1.72)	1.18 (0.71, 1.97)	1.05 (0.54, 2.01)
**Distance to major road** (m)			
> 200	*Reference*	*Reference*	*Reference*
150-200	1.46 (0.49, 4.38)	1.50 (0.84, 2.69)	0.54 (0.24, 1.20)
100-150	1.08 (0.33, 3.58)	0.95 (0.52, 1.76)	0.80 (0.42, 1.56)
50-100	0.94 (0.31, 2.89)	1.55 (0.90, 2.67)	0.95 (0.50, 1.79)
0-50	0.67 (0.18, 2.53)	1.44 (0.79, 2.63)	0.54 (0.25, 1.14)

[a] *Values listed are the <25th, 25-50th, 50-75th and >75th percentiles of the DWTD values.*

[b] *Values are odds ratios (95% confidence interval) and reflect the risk for small size for gestational age at birth for change in traffic parameters. Models are adjusted for fetal sex, maternal age, maternal ethnicity, maternal body mass index, parity, maternal smoking, maternal alcohol consumption, month of birth, and year of birth.*
