# Supplementary material for: Residential traffic exposure and pregnancy-related outcomes: a prospective birth cohort study
Source: Environ Health. 2009 Dec 22;8:59. doi: 10.1186/1476-069X-8-59 (PMC2811104; doi:10.1186/1476-069X-8-59)
Supplement: Additional file 7 — Table S7. Covariate-adjusted associations between residential traffic exposure and pregnancy-induced hypertension, stratified for maternal education. The table contains the results from the stratified analyses by educational level on the association between proximity to traffic and pregnancy-induced hypertension. [file 1476-069X-8-59-S7.PDF]

Additional file 7. Table S7. Covariate-adjusted associations between residential traffic exposure and pregnancy-induced hypertension, stratified for maternal education.

	**Pregnancy-induced hypertension** [b]		
	**None/primary education**	**Secondary education**	**Higher education**
**Distance-weighted traffic density** (veh/24h*m) [a]			
< 158,503	*Reference*	*Reference*	*Reference*
158,503 – 546,770	0.44 (0.03, 6.15)	1.38 (0.79, 2.40)	0.86 (0.50, 1.48)
546,770 – 1,235,384	6.00 (0.92, 39.21)	1.06 (0.60, 1.84)	0.62 (0.34, 1.13)
> 1,235,384	3.64 (0.60, 22.07)	1.32 (0.77, 2.29)	0.82 (0.48, 1.40)
**Distance to major road** (m)			
> 200	*Reference*	*Reference*	*Reference*
150-200	0.54 (0.05, 5.74)	1.25 (0.70, 2.26)	1.07 (0.59, 1.97)
100-150	2.56 (0.44, 15.06)	1.10 (0.64, 1.89)	0.66 (0.34, 1.29)
50-100	4.12 (0.92, 18.49) †	1.29 (0.75, 2.23)	0.82 (0.45, 1.50)
0-50	1.06 (0.17, 6.68)	0.66 (0.32, 1.35)	0.94 (0.53, 1.68)

† $p < 0.10$

[a] *Values listed are the <25th, 25-50th, 50-75th and >75th percentiles of the DWTD values.*

[b] *Values are odds ratios (95% confidence interval) and reflect the risk for pregnancy-induced hypertension for change in traffic parameters. Models are adjusted for maternal age, maternal ethnicity, maternal body mass index, parity, maternal smoking, maternal alcohol consumption, month of birth, and year of birth.*
