# Supplementary material for: Residential traffic exposure and pregnancy-related outcomes: a prospective birth cohort study
Source: Environ Health. 2009 Dec 22;8:59. doi: 10.1186/1476-069X-8-59 (PMC2811104; doi:10.1186/1476-069X-8-59)
Supplement: Additional file 8 — Table S8. Covariate-adjusted associations between residential traffic exposure and birth outcomes in non-movers (n = 1,118). The table presents the results from the sensitivity analyses on birth outcomes in the subgroup of non-movers. [file 1476-069X-8-59-S8.PDF]

Additional file 8. Table S8. Covariate-adjusted associations between residential traffic exposure and birth outcomes in non-movers (N=1,118).

	**Birth weight** (g) [b]	**Small for gestational age** [c]	**Preterm birth** [c]
**Distance-weighted traffic density** (veh/24h*m) [a]			
< 158,503	*Reference*	*Reference*	*Reference*
158,503 – 546,770	-1 (-73, 71)	0.84 (0.35, 2.05)	1.24 (0.58, 2.64)
546,770 – 1,235,384	36 (-37, 110)	0.92 (0.38, 2.22)	1.28 (0.59, 2.77)
> 1,235,384	40 (-33, 113)	0.57 (0.22, 1.51)	1.30 (0.61, 2.80)
**Distance to major road** (m)			
> 200	*Reference*	*Reference*	*Reference*
150-200	-51 (-132, 31)	1.59 (0.62, 4.05)	1.29 (0.59, 2.81)
100-150	-78 (-154, -3) *	1.03 (0.40, 2.62)	1.11 (0.53, 2.33)
50-100	6 (-70, 80)	1.11 (0.43, 2.85)	0.84 (0.36, 1.93)
0-50	-35 (-115, 45)	0.82 (0.28, 2.43)	1.30 (0.61, 2.79)

* $p < 0.05$

[a] *Values listed are the <25th, 25-50th, 50-75th and >75th percentiles of the DWTD values.*

[b] *Values are regression coefficients (95% confidence interval) and reflect the difference in birth weight for change in traffic parameters. The model is adjusted for gestational age, fetal sex, maternal age, maternal education, maternal ethnicity, maternal body mass index, parity, maternal smoking, maternal alcohol consumption, month of birth, and year of birth.*

[c] *Values are odds ratios (95% confidence interval) and reflect the risk for adverse birth outcomes for change in traffic parameters. Models are adjusted for fetal sex, maternal age, maternal education, maternal ethnicity, maternal body mass index, parity, maternal smoking, maternal alcohol consumption, month of birth, and year of birth.*
