# Supplementary material for: Residential traffic exposure and pregnancy-related outcomes: a prospective birth cohort study
Source: Environ Health. 2009 Dec 22;8:59. doi: 10.1186/1476-069X-8-59 (PMC2811104; doi:10.1186/1476-069X-8-59)
Supplement: Additional file 9 — Table S9. Covariate-adjusted associations between residential traffic exposure and pregnancy-complications in non-movers (n = 1,118). The table presents the results from the sensitivity analyses on pregnancy complications in the subgroup of non-movers. [file 1476-069X-8-59-S9.PDF]

Additional file 9. Table S9. Covariate-adjusted associations between residential traffic exposure and pregnancy complications in non-movers (N=1,118).

	**Pregnancy-induced hypertension** [b]	**(Pre)eclampsia or HELLP** [b]	**Gestational diabetes** [b]
**Distance-weighted traffic density** (veh/24h*m) [a]			
< 158,503	*Reference*	*Reference*	*Reference*
158,503 – 546,770	1.08 (0.39, 3.04)	0.75 (0.15, 3.69)	NA [c]
546,770 – 1,235,384	0.79 (0.25, 2.46)	1.95 (0.51, 7.41)	NA [c]
> 1,235,384	1.42 (0.51, 3.90)	1.95 (0.52, 7.35)	NA [c]
**Distance to major road** (m)			
> 200	*Reference*	*Reference*	*Reference*
150-200	0.24 (0.03, 1.92)	0.73 (0.14, 3.86)	NA [c]
100-150	1.11 (0.38, 3.18)	1.10 (0.28, 4.26)	NA [c]
50-100	1.03 (0.36, 2.92)	0.58 (0.11, 3.14)	NA [c]
0-50	1.60 (0.59, 4.36)	2.18 (0.64, 7.36)	NA [c]

[a] *Values listed are the <25th, 25-50th, 50-75th and >75th percentiles of the DWTD values.*

[b] *Values are odds ratios (95% confidence interval) and reflect the risk for pregnancy complications for change in traffic parameters. Models are adjusted for maternal age, maternal education, maternal ethnicity, maternal body mass index, parity, maternal smoking, maternal alcohol consumption, month of birth, and year of birth.*

[c] *Odds ratios could not be computed because of zero cell counts.*
